# Remission of long-term hepatic and renal disease induced by HCV after direct-acting antivirals therapy

**DOI:** 10.1590/2175-8239-JBN-2019-0165

**Published:** 2020-10-05

**Authors:** Raissa M Arruda, Andrea D Batista, Norma A Filgueira, Izolda F Moura, Luis H Sette, Edmundo P Lopes

**Affiliations:** 1 Universidade Federal de Pernambuco, Centro de Ciências Médicas, Recife, PE, Brasil.; 2 Universidade Federal de Pernambuco, Departamento de Medicina Clínica, Recife, PE, Brasil.; 3 Universidade Federal de Pernambuco, Hospital das Clínicas, Recife, PE, Brasil.

**Keywords:** Hepacivirus, Glomerulonephritis, Sofosbuvir, Simeprevir, Cryoglobulinemia, Hepacivirus, Glomerulonefrite, Sofosbuvir, Simeprevir, Crioglobulinemia

## Abstract

In addition to liver disease, the hepatitis C virus (HCV) has been associated with autoimmune phenomena, such as mixed cryoglobulin and glomerulonephritis (GN). Until recently, both chronic hepatitis and HCV extra-hepatic manifestations were treated with peg-interferon plus ribavirin, however these drugs presented low efficacy and induced severe side effects. Nowadays, the HCV chronic hepatitis has been treated with direct acting antivirals (DAA), but studies on the DAA therapy for HCV-associated glomerulonephritis are scarce. Here, we describe two cases of HCV-associated glomerulonephritis that were treated with DAAs. In these two cases, previously experienced to peg-interferon plus ribavirin, the sofosbuvir plus simeprevir therapy was effective, without significant side effects, and interrupted the evolution of at least 20 years of both hepatic and renal diseases. These cases join the seven previously described cases that were treated with this DAAs association.

## Resumo

Além da doença hepática, o vírus da hepatite C (HCV) tem sido associado a fenômenos autoimunes, como crioglobulinemia mista (CM) e glomerulonefrite (GN). Até recentemente, a hepatite crônica e as manifestações extra-hepáticas do HCV eram tratadas com peg-interferon com ribavirina; no entanto, essas drogas apresentavam baixa eficácia e induziam efeitos colaterais graves. Atualmente, a hepatite crônica por HCV tem sido tratada com antivirais de ação direta (AAD), mas estudos sobre a terapia com AAD para glomerulonefrite associada ao HCV são escassos. Aqui, descrevemos dois casos de glomerulonefrite associada ao HCV que foram tratados com AAD. Nestes dois casos, previamente tratados com peg-interferon e ribavirina, a terapia com sofosbuvir com simeprevir foi eficaz, sem efeitos colaterais significativos, e interrompeu a evolução de pelo menos 20 anos de doenças hepáticas e renais. Esses casos se juntam aos sete casos descritos anteriormente que foram tratados com essa associação de AAD.

## Introduction

Hepatitis C virus (HCV) infection affects approximately 71 million people, accounting for 400,000 deaths per year worldwide^1^. HCV infection causes high morbimortality, leading to liver cirrhosis and hepatocellular carcinoma in 4 to 20% of those infected^1^^,^ 2. In addition to liver disease, this virus has been associated with autoimmune phenomena, involving cryoglobulinemia, linfoproliferative disorders, and glomerulonephritis^2^^,^ 3.

Initially, HCV patients with glomerulopathies, in particular membrano- proliferative glomerulonephritis (MPGN), were treated with interferon (IFN) or peg-interferon (PEG-IFN) associated with ribavarin (RBV), but this therapy induced serious side effects and low sustained virologic response (SVR), often less than 50%^3^^,^ 4. The new direct-acting antivirals (DAAs) treatment are being considered revolutionary antiviral therapy, leading to infection cure by SVR in more than 90% of patients, without important side effects^3^^,^ 5. 

Although the first report of IFN-free regimen for patients with HCV-associated glomerulopathy was published in 1996, with RBV isolated use^6^, there are few reports on DAAs treatment in HCV patients with extra-hepatics manifestations, as glomerulopathies^3^^,^ 4. Because the isolated use of RBV promotes only transient viral HCV replication effects, since 2016 new reports have emerged using DAAs (IFN-free regimen) in HCV-associated glomerulopathies patients. Indeed, Saadoun et al. described five patients with HCV-associated glomerulopathies treated with sofosbuvir (SOF). Plus RBV for 24 weeks who achieved SVR^7^.

This study aimed to describe the treatment of two patients with chronic HCV infection associated to cryoglobulinemia and glomerulopathy, who were treated with the new DAAs SOF plus simeprevir (SIM) and achieved SVR. The two patients signed an informed consent form for the publication of these descriptions.

## Case Description

Case 1 - A 41-year-old man with diagnosis of chronic HCV infection associated with glomerulopathy has been followed in the reference services of Gastroenterology and Nephrology for 21 years. In 1997, with 20 years of age, he presented anasarca, creatinine clearance of 82.4 mL/min/1.73m^2^, proteinuria of 6.72 g/24h, positive cryoglobulinemia and positive HCV-RNA, being diagnosed by histopathological analysis with chronic hepatitis, with minimal inflammatory changes and mesangial proliferative glomerulonephritis. Initially, diuretics were introduced, and after edema regression, he was treated with IFN-α plus RBV for 24 weeks, evolving to aminotransferases normalization, HCV-RNA negativity, and proteinuria improvement. However, a few months later he presented viral relapse. He remained stable for some years, but in 2005 was admitted to the Nephrology service with anasarca and increased proteinuria. On this occasion, HCV genotype 1b infection was confirmed by PCR and new liver biopsy showed a METAVIR score of A1F1. This time, he received PEG-IFN plus RBV, but the treatment was discontinued due to therapeutic failure after 24 weeks. In 2016, he presented viral load of 2,064,684 UI/mL and log 6; serum creatinine was 1.1 mg/dL, proteinuria was 3.4 g/24h, and point shear wave elastography (pSWE) that shown 1.7 m/s (METAVIR ≈ F0). He was treated with SOF (400 mg/day) plus SIM (150 mg/day) for 12 weeks, evolving to normalization of aminotransferases and HCV-RNA negativity at the end of treatment, and after 12 weeks (SVR), cryoglobulinemia negativity and significant proteinuria reduction were obtained. The patient progresses asymptomatic and is being followed-up by the Nephrology service. 

Case 2 - A 50-year-old male was diagnosed 24 years ago with HCV infection prior to blood donation, and was referred to a Hepatology service. In 1995, he underwent hepatic biopsy that revealed persistent chronic hepatitis. He missed outpatient follow-up and returned in 2005, when genotype 1b infection was identified. He started treatment with PEG-IFN plus RBV, which was stopped after 24 weeks due to no virology response. He was followed-up on an outpatient basis, and in 2011 had a viral load of 791,479 IU/mL (log 5.9), when a new liver biopsy showed a METAVIR score of A2F3. In 2014, he presented a viral load of 1,193,977 IU/mL (log 6.08), evolving to thrombocytopenia, hypoalbuminemia, ascites, and decreased renal function, in addition to hematuria and hematic casts. He was diagnosed with HCV-associated glomerulopathy, but a renal biopsy was not possible due to thrombocytopenia. He presented C3 and C4 consumption, creatinine clearance of 65 mL/min/1.73m^2^, proteinuria of 4.33 g/24h, and positive cryoglobulinemia. Spironolactone and furosemide were prescribed with edema improvement. In 2015, he performed pSWE, which revealed 3.01 m/s (METAVIR ≈ F4). In 2016, he was treated with SOF (400 mg/day) plus SIM (150 mg/day) for 12 weeks, evolving with transaminases normalization, HCV-RNA negativity at the end of treatment, and after 12 weeks (SVR), cryoglobulinemia negativity and significant reduction of proteinuria were obtained. He is asymptomatic but continues to be followed-up in Hepatology and Nephrology services.

## Discussion

Starting in 2014, the first cases of DAA use with the NS3-4A proteases inhibitors (boceprevir and telaprevir) in HCV-associated glomerulopathy therapy were published, and clinical improvement after HCV eradication was described. Nevertheless, these first-generation DAAs were always administered in association with PEG-IFN/RBV and used for long periods, triggering serious side effects, mainly severe anemia^8^^,^ 9 ^,^ 10.

In the following year, 2015, a study described the first two cases of HCV-associated glomerulopathies treated with second-generation treated with second-generation DAAs, associating SOF with PEG/RBV for 12 weeks, associated to PEG/RBV for 12 weeks, and both cases showed clinical improvement after SVR^9^.

Then, in 2016, the cases of five more patients with HCV-associated glomerulopathy treated for 24 weeks with a truly IFN-free regimen, using SOF and RBV, were described. All achieved SVR and four presented renal disease improvement^7^. After, 12 patients with HCV-associated mixed cryoglobulinemia syndrome (MCS) were described, seven of them with glomerulopathies. The history of MCS ranged from 1 to 17 years. Among these seven patients, six were treated with SOF plus SIM and the other one, which presented genotype 2, with SOF plus RBV. Six of the seven patients achieved not only SVR, but also glomerular filtration rate and proteinuria improvement^11^. A previous study reported a series of 44 patients with mixed HCV-associated cryoglobulinemia, of which four (9%) also had glomerulopathies with reduced glomerular filtration rate and proteinuria. Three were treated with SOF plus RBV associated with SIM, or daclatasvir (DAC), or ledipasvir (LED), and one patient with SOF plus RBV. All these four patients achieved SVR, in addition to improved glomerular filtration rate and proteinuria^12^.

In 2017, a patient with HCV-associated membranous glomerulopathy, genotype 1b, who was treated with SOF plus LED for 12 weeks achieved SVR at week 12 with glomerular filtration rate and proteinuria improvement^13^. Subsequently, a series of 18 patients with MCS was described, of which 10 (55%) had renal involvement. All of them were treated with DAAs with and without PEG-IFN and seven achieved SVR^14^. Another HCV-associated MCS patient, genotype 3a, was treated with SOF plus DAC for 24 weeks. He achieved SVR with glomerular filtration rate and proteinuria improvement, allowing antihypertensive and diuretic suspension. However, the patient persisted with cryoglobulinemia and microscopic hematuria^15^.

Also in 2017, a series of 41 patients with HCV-associated MCS was described, of which five patients (12%) also had renal involvement, four of them with MPGN. All five patients were treated with SOF plus DAC for 12 or 24 weeks, achieving SVR and proteinuria improvement, and hematuria disappeared in four of five cases at week 24^16^.

Then, a series of 35 patients with HCV-associated MCS was described, of which seven patients (20%) had renal involvement. All seven were treated with DAAs and achieved SVR, in addition to glomerular filtration rate improvement, proteinuria decrease, and hematuria disappearance^17^. Another series of 22 patients with MCS associated with HCV, of which four patients (18%) had cryoglobulinemia glomerulonephritis and were treated with DAAs, all achieving SVR^18^.

In 2018, a series of 63 patients with HCV-associated MCS, seven of which (11%) had glomerulonephritis, was published. All of them were treated with SOF plus DAC for three months, achieved SVR and cryoglobulin disappearance,e and five also showed renal disease improvement^19^.

More recently, the therapy with ombitasvir-paritaprevir-ritonavir plus dasabuvir for 12 weeks of 9 patients with mixed HCV-associated MCS and glomerulonephritis was reported. All the patients presented SVR, but only two had complete clinical recovery, and six presented persistent or worsening of MCS and received immunosuppressive therapy^20^.

The analysis of the new DAAs therapy efficacy was described in the review article by Fabrizi et al., (2019), revealing that SVR can be achieved in 95% (297 of 313) of patients with HCV-associated MCS^3^. These results are similar to those observed in the literature with the DAAs use in the HCV treatment of patients without MCS^5^. Among these 313 patients, 52 (17%) had MCS with renal involvement, of which 49 (94%) achieved SVR, 17 (33%), complete GN resolution, and 15 (29%), kidney disease improvement^3^. The two new cases reported in this paper confirm DAAs high efficacy in the HCV-associated MCS treatment with renal involvement, since both achieved SVR, cryoglobulinemia negativity, and proteinuria improvement after the SOF plus SIM use. Nevertheless, some cases of new onset or relapsing MCS and glomerulonephritis in patients with HCV after successfully treated (SVR) with DAAs have been described^3^^,^ 20. In these relapse cases, immunosuppressive agents, such as rituximab, could be used^3^^,^ 20.

In conclusion, in these two cases of cryoglobulinemia and renal involvement associated with HCV, previously experienced to PEG-IFN plus RBV, the SOF plus SIM therapy was effective, without significant side effects, and interrupted the evolution of at least 20 years of both hepatic and renal disease.
